# School refusal behavior in children and adolescents: a concept analysis

**DOI:** 10.3389/fpsyt.2026.1954084

**Published:** 2026-09-18

**Authors:** Luoyan Wang, Xiang Li, Xiaodi Li, Mengxiao Lei, Hong Luo, Bowen Xue

**Affiliations:** 1 Affiliated Mental Health Center & Hangzhou Seventh People’s Hospital, Zhejiang University School of Medicine, Hangzhou, China; 2 Research Center for Mental Health and Humanities, Zhejiang University School of Medicine, Hangzhou, China; 3 School of Nursing, Hangzhou Normal University, Hangzhou, China; 4 Department of Psychology and Behavioral Sciences, Zhejiang University, Hangzhou, China

**Keywords:** children and adolescents, concept analysis, emotional distress, school absenteeism, school refusal behavior

## Abstract

School refusal behavior (SRB) is increasingly recognized across educational, pediatric, and mental health settings, yet its conceptual boundaries remain unclear, and the term is frequently conflated with school refusal, truancy, school withdrawal, school exclusion, and broader school attendance problems. This study used Walker and Avant’s concept analysis method to clarify the meaning, defining attributes, antecedents, consequences, and empirical referents of SRB. PubMed, Web of Science, Scopus, and PsycINFO were searched for English-language literature published through January 2026, supplemented by Google Scholar and manual citation searching. A total of 100 sources were included and analyzed through iterative comparison of conceptual definitions, recurrent characteristics, contextual conditions, outcomes, and indicators used in research and practice. The analysis showed that SRB has evolved from earlier anxiety-centered descriptions toward a broader behavioral and functional construct. Four defining attributes were identified: difficulty initiating, sustaining, or completing school attendance or participation; young person–expressed attendance difficulty; aversive school-related experiences or emotional distress; and heterogeneous avoidance- or reinforcement-related maintaining functions. Antecedents were distributed across individual, family, peer and interpersonal, and school-contextual domains, while consequences extended across academic, psychological, social, family, and service-related domains and may accumulate over time. Empirical identification requires the integration of observable attendance patterns, emotional, behavioral, and somatic responses, functional assessment, and contextual information from young people, caregivers, and school personnel. Moving beyond the summary of existing definitions, this analysis proposes an integrative framework that distinguishes the observable form of attendance difficulty from its emotional presentation, maintaining functions, and ecological context. SRB is therefore best understood as a persistent or recurrent, young person–expressed pattern of disrupted school attendance or participation that is clinically meaningful but cannot be reduced to a single diagnosis, cause, or attendance threshold. By clarifying what constitutes SRB and how it differs from other forms of school attendance problems, this framework may support earlier recognition of emerging attendance difficulties, more comprehensive assessment of underlying needs, and better coordination among mental health professionals, families, and schools.

## 1 Introduction

In recent years, school refusal behavior (SRB) among children and adolescents has attracted increasing attention across the fields of education, developmental psychology, and public health. In real-world contexts, SRB rarely presents as a simple and absolute form of complete school nonattendance. Rather, it often unfolds as a gradual and heterogeneous process, including repeated tardiness, frequent absenteeism, arriving at school but struggling to enter the classroom, persistent requests to leave school during the day, or marked avoidance responses during examinations and other stressful school situations (1). The estimated detection rate of SRB among adolescents is approximately 31.9%, suggesting that SRB is not a rare or isolated phenomenon but may have substantial public health significance (2). Existing studies have shown that SRB is associated with reduced academic engagement, lower school belonging, and poorer perceptions of safety and meaning in school experiences (3). Moreover, persistent SRB has been associated with the risk of school dropout and subsequent mental health problems; it may also place considerable strain on families and contribute to broader social and service burdens (4, 5).

At the same time, SRB remains embedded in a broader and often confusing set of school attendance–related terms. In the literature, school refusal generally refers to situations in which children or adolescents refuse to attend school or experience marked difficulty remaining in school because of emotional distress, with parental awareness and without severe antisocial behavior (6). Closely related but somewhat broader in scope, school refusal behavior is often used to describe a pattern in which children or adolescents voluntarily refuse to attend school and/or have difficulty remaining in school for the entire school day (7). By contrast, truancy usually refers to unexcused absence concealed from parents and more often associated with antisocial behavior or rule violations (8). School withdrawal refers to school absence primarily driven by parental decisions or family-related circumstances, whereas school exclusion denotes school-initiated removal from regular educational activities for disciplinary or administrative reasons (9, 10). To integrate these diverse attendance-related phenomena, some scholars have proposed the broader term school attendance problems (SAPs), which encompasses various forms of difficulty attending or participating in school arising from emotional, behavioral, family, health-related, and school-contextual factors (9, 11). Within this broader framework, SRB represents a distinct pattern characterized by young person–expressed difficulty in attending or remaining engaged in school, particularly when attendance problems are associated with school-related emotional distress and avoidance-related functions. Recent research has further emphasized that emotional discomfort and affective experiences are central components of school refusal, helping to differentiate SRB from other forms of school attendance problems (12). In adolescent populations, school non-attendance has also been increasingly conceptualized as a multifaceted phenomenon involving psychological, social, and educational vulnerabilities rather than a single behavioral outcome (13). However, these terms are frequently used interchangeably or inconsistently across studies, contributing to conceptual ambiguity in the school attendance literature.

The conceptual instability surrounding SRB is also rooted in its historical development. Earlier research was largely shaped by diagnostic-oriented perspectives on school refusal, emphasizing emotional distress, dependence on home, and the absence of severe conduct problems (11). Later work shifted toward a functional understanding of SRB, focusing on the different maintaining mechanisms underlying school avoidance, such as avoiding negative affectivity, escaping social or evaluative situations, seeking attention from significant others, or obtaining tangible reinforcement outside school (14). Contemporary perspectives have increasingly moved beyond anxiety-centered explanations by emphasizing that school attendance difficulties emerge through dynamic interactions among young people’s experiences, relationships, and educational contexts, including family processes, peer experiences, and school conditions (9, 15, 16). Recent systematic reviews and bibliometric analyses have further documented this expanding conceptual landscape, showing that SRB research has increasingly incorporated psychological, interpersonal, educational, and contextual perspectives beyond early clinical descriptions (17, 18).

As a result, the core meaning and conceptual boundaries of SRB remain insufficiently clarified (6, 19). This ambiguity is not merely terminological. Equating SRB with anxiety-based school refusal or general absenteeism may obscure differences in emotional, behavioral, somatic, and contextual presentation. Clinically meaningful difficulties may be missed when distress appears as irritability, physical complaints, partial attendance, or difficulty remaining in class rather than complete absence. Conversely, viewing SRB through a single diagnostic lens may conceal the developmental, interpersonal, family, and school processes that shape it. Conceptual imprecision also weakens comparability across studies, complicates assessment and intervention, and impedes communication across educational, mental health, and medical systems. A coherent account is therefore needed that preserves the clinical significance of SRB without reducing it to one symptom, diagnosis, cause, or attendance threshold. Accordingly, this study clarifies its defining attributes, antecedents, consequences, and empirical referents, distinguishes it from related attendance problems, and proposes an integrative framework for early recognition, differential assessment, individualized formulation, and coordinated care.

## 2 Methods

### 2.1 Study design

This study employed the concept analysis method proposed by Walker and Avant to clarify the concept of school refusal behavior (SRB). Concept analysis is a theory-oriented and literature-based approach that aims to examine how a concept is used in existing scholarship, identify its defining attributes, and distinguish it from related constructs. Because the present study focused on conceptual clarification based on published literature rather than primary data collection, no human participants were involved. To enhance the transparency of the literature identification and selection process, the search and screening procedures were reported with reference to the Preferred Reporting Items for Systematic Reviews and Meta-Analyses (PRISMA) guidelines (20).

### 2.2 Literature search

A systematic literature search was conducted to identify studies relevant to the concept of school refusal behavior (SRB). Four electronic databases were searched, namely PubMed, Web of Science, Scopus, and PsycINFO. The main search terms included “school refusal,” “school refusal behavior,” and “school refusal behaviour,” which were combined with population-related terms such as child*, adolescent*, youth, and student*. The search included studies published up to January 2026. To enhance comprehensiveness, additional searches were conducted through Google Scholar and manual citation searching of the reference lists of relevant articles. The detailed search strategy is provided in **Supplementary Material S1**.

The database search yielded 1,398 records, including 338 from PubMed, 310 from Web of Science, 656 from Scopus, and 94 from PsycINFO. After removal of 278 duplicate records, 1,120 records remained for screening. Following title and language screening, 302 records were excluded, leaving 818 records for abstract review. After abstract screening, 146 full-text articles were assessed for eligibility. Of these, 65 articles were excluded, including 14 due to inappropriate study populations and 51 because their focus was only weakly or indirectly related to the SRB concept. In addition, 22 records were identified through supplementary sources, including website searching and citation tracking; among these, 19 studies were eligible for inclusion after screening. The literature identification, screening, eligibility assessment, and inclusion process were documented in a PRISMA 2020 flow diagram (**Figure 1**).

**Figure 1:**
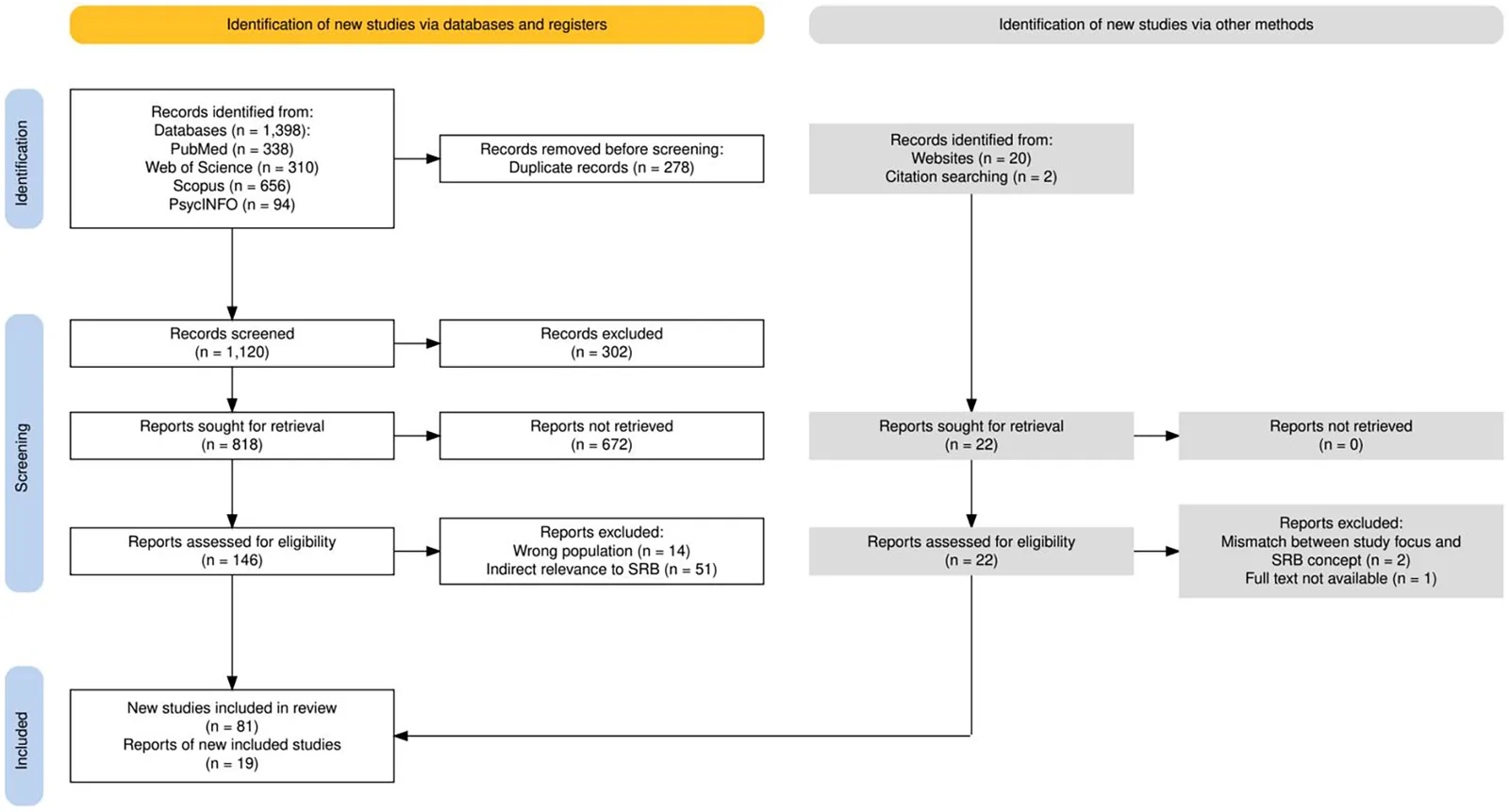
PRISMA 2020 flow diagram of study selection.

### 2.3 Inclusion and exclusion criteria

Studies were included if they met the following criteria: (a) focused on school refusal behavior or closely related school attendance constructs relevant to the conceptualization of SRB; (b) contributed to the definition, characteristics, conceptual boundaries, antecedents, consequences, or empirical identification of SRB; (c) were qualitative, quantitative, mixed-methods, theoretical, or review articles; (d) were published in peer-reviewed journals; and (e) were published in English up to January 2026.

Studies were excluded if SRB was not a central focus of the article, if the study population was not relevant to the present concept analysis, or if the publication was a study protocol, conference abstract, editorial, commentary, or opinion paper that did not provide sufficient conceptual information for analysis. In addition, studies that were only indirectly related to the SRB concept were excluded during the full-text eligibility assessment.

### 2.4 Data extraction and analytic procedure

A structured data extraction matrix was developed for this study and used to summarize the included literature in a consistent manner. For each study, the extracted information included bibliographic identification, study type, definitional statements related to school refusal behavior (SRB), core attributes of the concept, antecedents, consequences, theoretical framework, and additional analytic notes relevant to conceptual clarification. The purpose of this extraction process was not merely to summarize study findings, but to capture how SRB was defined, characterized, and differentiated across the literature.

The analytic process was guided by Walker and Avant’s concept analysis approach. First, the uses of the concept were examined across the included studies, including historical and contemporary terminology related to school refusal, school refusal behavior, and adjacent school attendance constructs. Second, recurrent descriptions and characteristics were compared across studies to identify the defining attributes of SRB. Third, antecedents and consequences reported in the literature were extracted and grouped into broader thematic categories. Fourth, empirical referents were identified to clarify how SRB may be recognized and operationalized in research and practice. Throughout the analytic process, conceptually similar content was repeatedly compared, merged, and refined in an iterative manner until a clearer conceptual structure emerged.

Based on this iterative comparison, the final synthesis focused on clarifying the conceptual boundaries of SRB and distinguishing it from related constructs such as truancy, school withdrawal, school exclusion, and broader school attendance problems. The results of the analysis were then organized around the main components of concept analysis, including the uses of the concept, defining attributes, antecedents, consequences, empirical referents, and the development of an integrative working definition of SRB.

## 3 Results

### 3.1 Uses of the concept

Across the included literature, the phenomenon now commonly referred to as school refusal behavior (SRB) did not emerge as an independent concept from the outset, but developed gradually from earlier labels such as school phobia and school refusal (21, 22). This conceptual progression reflects a broader shift in how attendance-related difficulties have been understood: from a relatively narrow anxiety-based interpretation toward a more behavioral, heterogeneous, and functionally oriented framework. In early discussions, related phenomena were often described as school phobia or school refusal, with primary attention given to children’s fear, distress, and separation-related difficulties when facing school attendance (21). Within this early framework, the problem was largely conceptualized as an emotionally based clinical phenomenon. However, as research accumulated, scholars increasingly recognized that not all young people with school attendance difficulties could be adequately understood as simply “afraid of school.” In many cases, the central difficulty appeared to involve intolerance of social evaluation, academic failure, separation from caregivers, or other distressing school-related experiences rather than fear of school itself (22, 23).

Against this background, school refusal gradually replaced school phobia as a broader and less pathologizing descriptive term. Compared with school phobia, school refusal shifted the focus from a presumed fear-based disorder to the observable phenomenon of difficulty attending school, while still retaining core features such as emotional distress, parental awareness, and the relative absence of severe antisocial behavior (8, 11, 22). Nevertheless, school refusal remained largely descriptive and offered limited explanatory power regarding why the behavior occurred, why its manifestations varied across individuals, and how it was maintained over time. This limitation helped create space for later formulations that emphasized heterogeneity and functional differences within attendance-related difficulties (22, 23).

The introduction of school refusal behavior marked a further conceptual development. Relative to school refusal, SRB more explicitly frames the phenomenon as a behavioral construct. Its focus extends beyond complete nonattendance to a broader continuum of school attendance difficulty, including lateness, partial absence, leaving school during the day, difficulty entering the classroom, and prolonged absence (4, 10). Importantly, SRB also incorporates the possibility that these behaviors may be maintained by different underlying functions. In this regard, Kearney’s functional model played a central role in shaping contemporary usage of the term, highlighting that SRB may serve functions such as avoiding negative affectivity, escaping social or evaluative situations, seeking attention from significant others, or obtaining tangible reinforcement outside school (14). As a result, SRB became increasingly prominent in functional assessment studies, profile-based research, and quantitative investigations of school attendance difficulty (4, 23).

At the same time, the literature contains several adjacent expressions, including anxiety-based school refusal and emotionally based school avoidance, indicating that emotional distress remains widely recognized as a central component of the phenomenon even as behavioral and functional interpretations have expanded (10, 23, 24). In parallel, SRB has increasingly been discussed within broader frameworks such as school absenteeism, school non-attendance, and especially school attendance problems (SAPs) (9, 25). Within this broader terminology, SRB is generally treated not as a synonym for all forms of absence, but as one specific subtype of attendance difficulty. Most studies also continue to distinguish SRB from truancy, with the latter being more strongly associated with concealment, oppositionality, and externalizing or antisocial behavior, whereas SRB is more often characterized by school-related distress, parental awareness, and relatively limited severe conduct problems (8–10). Similarly, school withdrawal and school exclusion are conceptually differentiated from SRB because the primary initiating agent lies in the family or school system rather than in the young person’s own distress-linked avoidance of attendance (9, 10, 25).

Overall, the uses of the concept suggest a clear developmental trajectory from the anxiety-centered label of school phobia, to the descriptive term school refusal, and finally to the more inclusive and analytically useful construct of school refusal behavior. Compared with earlier terminology, SRB better captures the continuum, heterogeneity, and multiple maintaining processes of attendance-related difficulty (10, 14, 23). Conceptually, this makes SRB more suitable than narrower earlier terms for a contemporary concept analysis, because it allows the phenomenon to be examined not only as an emotionally based attendance problem, but also as a behavior pattern shaped by diverse functions and contexts. For this reason, the present analysis treats SRB as the most appropriate focal concept for clarifying the defining attributes, antecedents, consequences, and empirical referents of this phenomenon.

### 3.2 Defining attributes

According to Walker and Avant, defining attributes are the characteristics that appear repeatedly in the literature and help distinguish a concept from related phenomena. Across the included studies, school refusal behavior (SRB) was not represented as a single uniform act of “not going to school,” but rather as a heterogeneous pattern of school attendance difficulty linked to emotional distress and maintained by different underlying processes. Four defining attributes were identified: (1) difficulty attending and/or remaining in school, (2) young person–expressed attendance difficulty (3) emotional distress associated with school attendance, and (4) functional heterogeneity in the maintenance of the behavior (9, 10, 14).

#### 3.2.1 Difficulty attending and/or remaining in school

The most consistently reported attribute of SRB is difficulty attending school and/or remaining in school once attendance has begun. Importantly, this difficulty is not limited to complete school nonattendance. Rather, the literature indicates that SRB may be expressed along a continuum, including repeated lateness, partial absence, reluctance to enter the classroom, requests to leave school during the day, intermittent attendance, and prolonged absence (1, 4, 10). This feature is conceptually important because it positions SRB as a pattern of disrupted school participation rather than a simple binary state of attendance versus nonattendance. In other words, SRB refers not only to whether a student attends school, but also to whether the student can tolerate, sustain, and engage in school attendance across time.

#### 3.2.2 Young person–expressed attendance difficulty

A second defining attribute is that the attendance difficulty is experienced and expressed through the young person. In the literature, SRB is distinguished from school withdrawal and school exclusion precisely because the primary initiating force in those constructs lies outside the student, namely in parental decisions or school-imposed actions (8–10). By contrast, SRB refers to a pattern in which the child or adolescent experiences difficulty initiating, sustaining, or remaining in school, often in the context of emotional distress and other school-related pressures. Such attendance difficulty may involve avoidance or resistance without necessarily reflecting a straightforward or freely chosen decision not to attend. Broader family, peer, school, and contextual factors may also shape the circumstances in which attendance becomes difficult to sustain. Conceptually, this attribute is central because it helps preserve the boundary between SRB and other school attendance problems in which absence is primarily initiated by parents or imposed by institutions.

#### 3.2.3 Emotional distress associated with school attendance

A third defining attribute is the presence of emotional distress linked to school attendance. Across conceptual, review, and empirical literature, SRB is repeatedly associated with anxiety, fear, sadness, somatic complaints, separation-related discomfort, or marked tension in anticipation of attending school (15, 16, 23, 26). Emotional distress does not mean that every young person with SRB necessarily meets criteria for an anxiety disorder, depressive disorder, or other psychiatric diagnosis. Rather, it indicates that school attendance is experienced as aversive, overwhelming, or emotionally dysregulating, and that this distress is central to the emergence and persistence of the behavior. Conceptually, this attribute is particularly important because it helps distinguish SRB from forms of absenteeism that are more strongly characterized by concealment, delinquency, or deliberate rule violation.

#### 3.2.4 Functional heterogeneity in the maintenance of the behavior

The fourth defining attribute is that SRB is functionally heterogeneous rather than etiologically uniform. The literature does not support understanding SRB as a single syndrome with one fixed cause. Instead, SRB may be maintained by different functional processes, including avoidance of school-based negative affectivity, escape from social or evaluative situations, pursuit of attention from significant others, or access to reinforcing experiences outside school (14, 27–29). This attribute is conceptually central because it explains why students with superficially similar attendance problems may differ substantially in motivation, clinical presentation, and intervention needs. It also clarifies why SRB is better conceptualized as a behavioral construct than as a narrowly defined diagnostic category.

Taken together, these findings suggest that school refusal behavior (SRB) is a distress-linked, young person–expressed, and functionally heterogeneous pattern of difficulty attending and/or remaining in school. It is distinguished from truancy, school withdrawal, and school exclusion by the nature and context of the young person’s attendance difficulty, rather than concealment, parental removal, or institutionally imposed absence.

### 3.3 Cases

In Walker and Avant’s approach, cases are used to clarify the conceptual boundaries of a phenomenon by illustrating what the concept is, what it is related to, and what it is not. In the present analysis, a model case, a borderline case, a related case, and a contrary case were constructed to further clarify the meaning and boundaries of school refusal behavior (SRB).

#### 3.3.1 Model case

A 13-year-old girl has experienced increasing difficulty attending school over the past three months. On school mornings, she reports intense anxiety, stomach pain, and fear of being called on in class. Her parents are fully aware of her distress and repeatedly attempt to encourage attendance. On some days, she arrives at school but is unable to enter the classroom; on other days, she asks to leave early because she feels overwhelmed. Her absences increase during examination periods and after episodes of peer embarrassment in class. She does not conceal her absence from her parents, shows no significant antisocial behavior, and expresses guilt about missing school. Assessment suggests that her attendance difficulty is maintained by avoidance of negative affect and escape from evaluative situations. This case demonstrates all defining attributes of SRB: difficulty attending and remaining in school, young person–expressed attendance difficulty emotional distress linked to school attendance, and functional heterogeneity.

#### 3.3.2 Borderline case

A 12-year-old boy has missed several days of school over the last month. He complains of headaches and fatigue on school mornings and sometimes refuses to attend. However, he has also recently been recovering from a documented medical condition, and his absences are partly related to medical appointments and parental caution. He reports some worry about school, but emotional distress is not consistently evident, and his attendance difficulty cannot be clearly distinguished from health-related absence. This case contains some features associated with SRB, particularly school attendance difficulty, but does not fully demonstrate the defining emotional and functional characteristics of SRB. It therefore represents a borderline case rather than a clear example of the concept.

#### 3.3.3 Related case

A 15-year-old boy frequently skips school and spends the day with peers away from home. He leaves home as if going to school, conceals his absence from his parents, and shows little evidence of school-related fear, anxiety, or distress. His absences are associated with oppositional behavior and rule violation rather than emotional discomfort related to school attendance. This case is related to SRB because it involves school nonattendance, but it is more consistent with truancy. It helps clarify that not all forms of absenteeism fall within SRB, especially when concealment, antisocial behavior, and absence of school-related distress are prominent.

#### 3.3.4 Contrary case

A 14-year-old student is suspended from school for repeated violent behavior toward classmates. During the suspension period, he does not attend school because the school has formally prohibited attendance. He expresses no avoidance, fear, or distress about school attendance, and the absence is determined by disciplinary action rather than by the student’s own emotionally linked difficulty attending school. This case is contrary to SRB because it lacks the defining attributes of school refusal behavior and instead reflects school exclusion.

Taken together, these cases further clarify that SRB is not equivalent to all forms of school absence. Rather, it refers specifically to a distress-linked, young person–expressed pattern of difficulty attending and/or remaining in school, which must be distinguished from mixed or medically influenced attendance problems, truancy, and school exclusion.

### 3.4 Antecedents

Across the included literature, factors associated with school refusal behavior (SRB) clustered across multiple ecological levels, indicating that SRB is associated with emotional, interpersonal, and contextual risks rather than with a single psychopathological pathway (16). For analytic clarity, these antecedents were grouped into four broad categories: individual vulnerabilities, family-related factors, peer and interpersonal difficulties, and school-contextual stressors.

#### 3.4.1 Individual vulnerabilities

At the individual level, emotional difficulties appear to be among the most frequently reported antecedents of SRB. Anxiety and anxiety-related symptoms are repeatedly identified as especially central, including school anxiety, social anxiety, separation-related distress, fear of negative evaluation, and somatic distress associated with school attendance (14, 28). Depressive symptoms, emotional dysregulation, and heightened stress reactivity have also been described as important individual-level vulnerabilities (28, 30). In addition, more recent evidence suggests that attentional problems and self-regulatory difficulties have been associated with SRB, with school anxiety and avoidance identified as potential statistical pathways underlying these associations (2). Taken together, these findings indicate that SRB often develops in young people who experience school as emotionally aversive, overwhelming, or difficult to regulate psychologically.

#### 3.4.2 Family-related factors

Family factors also emerged as important antecedents of SRB. The literature suggests that school refusal may be preceded by family conflict, unstable family relationships, overdependence within parent-child relationships, difficulties with separation, and limited emotional support at home (31). In qualitative research with Chinese adolescents, school refusal was described as developing and being maintained within broader family and social systems, including conflictual family dynamics, insufficient support resources, and difficulties in returning to school (15). Newer evidence further suggests that parental emotion dysregulation, parental anxiety and depression, and maladaptive rearing styles may be associated with adolescent SRB, indicating that family processes are not merely background conditions but active components in the development and maintenance of school avoidance (30).

#### 3.4.3 Peer and interpersonal difficulties

A third cluster of antecedents concerns peer and interpersonal functioning. Experiences such as bullying, peer rejection, social exclusion, and fear of social evaluation have been repeatedly linked to SRB (2, 15, 28). These factors appear particularly relevant for young people whose school attendance difficulties are tied to social anxiety, interpersonal sensitivity, or efforts to avoid humiliating or threatening peer situations. Recent adolescent evidence also suggests that experiences of being bullied have been associated with SRB, with school anxiety and related distress statistically accounting for part of this association (2). Conceptually, these interpersonal factors are important because they indicate that SRB is not only an individual emotional problem, but also a response to the social environment in which school attendance takes place (9).

#### 3.4.4 School and contextual stressors

School-related and broader contextual stressors constitute another major antecedent domain. Across the literature, academic pressure, learning difficulties, exam-related stress, low school belonging, negative attitudes toward school, and poor adaptation to school demands have all been associated with SRB (32, 33). Teacher behavior is also relevant: autonomy-supportive and respectful teaching practices may buffer against attendance difficulties, whereas psychologically controlling or pressure-based instructional styles have been associated with greater school refusal and absenteeism, as well as lower satisfaction of basic psychological needs (34). More broadly, recent evidence suggests that environmental adversity, including bullying, maltreatment, and other psychosocial stressors, may interact with school-based pressures and has been associated with more persistent or clinically significant forms of SRB (35). These findings suggest that antecedents of SRB are partly embedded in the everyday structure, demands, and emotional climate of school itself, where students’ experiences of academic, social, and emotional demands are shaped.

Overall, the antecedents of SRB are best understood as an interacting ecological network rather than a linear chain of causes. Individual emotional and regulatory characteristics have been associated with how young people experience academic and interpersonal demands, while family, peer, and school-contextual factors are also associated with distress, coping resources, and the conditions under which attendance is experienced as aversive or difficult to sustain (9).

### 3.5 Consequences of school refusal behavior

The consequences of school refusal behavior (SRB) extend well beyond school absence itself and can be observed at academic, psychological-social, family, and broader systemic levels. Rather than representing only a short-term attendance difficulty, SRB has been associated with adverse outcomes in students’ educational participation, emotional and social functioning, family well-being, and longer-term service needs (4, 16, 23).

#### 3.5.1 Academic consequences

One of the most immediate and consistently reported consequences of SRB is disruption to academic engagement and school participation. Repeated nonattendance, partial attendance, lateness, and difficulty remaining in school reduce students’ exposure to classroom learning and may weaken their sense of belonging and investment in school. Empirical studies have shown that SRB is associated with less adaptive academic self-attributions, lower academic self-efficacy, poorer academic adjustment, and a greater risk of sustained educational disengagement (3, 33, 34). Over time, persistent attendance difficulty has also been associated with chronic underachievement or school dropout (4).

#### 3.5.2 Psychological and social consequences

SRB may also be followed by substantial psychological and psychosocial difficulties. Young people with persistent school attendance problems often experience worsening anxiety, depressive symptoms, stress, social withdrawal, and reduced school belonging or perceived safety (35). Because school is a major developmental context for peer interaction, routine, competence building, and identity development, sustained withdrawal from school may further intensify loneliness, reduce opportunities for social participation, and reinforce maladaptive avoidance patterns. In this sense, SRB may function not only as a response to distress, but also as a process that maintains or amplifies emotional and interpersonal difficulties over time (35). This cyclical pattern is conceptually important because it suggests that the consequences of SRB may also become part of the conditions that sustain the behavior.

#### 3.5.3 Family-level consequences

At the family level, SRB is frequently associated with increased caregiver burden, parental stress, and disruption in family functioning. Families may experience repeated conflict around school attendance, emotional exhaustion related to daily efforts to encourage or manage attendance, and uncertainty about how to balance protection, support, and limit-setting. Qualitative and review evidence suggests that prolonged school absence and unsuccessful attempts at school re-entry may place sustained strain on parent-child relationships and family routines (15, 36). These family-level consequences indicate that SRB should not be understood solely as an individual student problem, but also as a phenomenon that affects the wider family system.

#### 3.5.4 Broader service and societal consequences

Beyond the student and family, persistent SRB may also increase demand on educational, psychological, and mental health support systems. When attendance difficulties become chronic, schools often require greater coordination with families, counselors, psychologists, and clinical services, and unsuccessful intervention may contribute to prolonged disengagement and higher long-term social costs (4, 23). Recent review literature further suggests that SRB commonly co-occurs with broader mental health and psychosocial difficulties, implying the need for multi-system intervention rather than single-setting management alone (35). Accordingly, the consequences of SRB may extend beyond absenteeism per se and influence later educational pathways, service utilization, and social adaptation.

Overall, the consequences of SRB suggest that it should not be regarded as a minor or temporary attendance irregularity. Available evidence indicates that, in some cases, SRB may become embedded in reciprocal patterns involving academic disengagement, emotional distress, family strain, and increased service needs. However, these patterns are heterogeneous and should not be interpreted as an inevitable consequence of continued non-attendance. This pattern further supports the importance of early identification and clear conceptual differentiation of SRB from other forms of school attendance problems, as associated difficulties may accumulate across developmental, relational, and institutional levels (9, 37).

### 3.6 Empirical referents

Empirical referents are the observable phenomena by which a concept can be recognized in research and practice (38). For school refusal behavior (SRB), no single indicator is sufficient on its own. Rather, SRB is best identified through a combination of observable attendance patterns, distress-related indicators, functional assessment, and multi-informant contextual information (9, 23).

#### 3.6.1 Observable attendance-related indicators

At the most visible level, SRB may be empirically reflected in repeated lateness, partial attendance, intermittent absence, prolonged absence, difficulty entering the school building or classroom, and requests to leave school during the day (1, 4). These indicators are important because they translate the defining attribute of difficulty attending and/or remaining in school into observable patterns of school participation. In practice, school attendance records, class absence records, and documented patterns of incomplete school participation can therefore serve as important empirical referents for identifying the behavioral expression of SRB.

#### 3.6.2 Distress-related indicators associated with school attendance

Because SRB is conceptually linked to emotional distress, empirical referents also include signs that school attendance is experienced as aversive, overwhelming, or emotionally dysregulating (23). Such signs may include anxiety before school, marked school-related fear, somatic complaints on school mornings, distress when separating from caregivers, or heightened emotional reactivity in anticipation of school attendance (26). These indicators are especially useful because they help distinguish SRB from school absence that is more strongly characterized by concealment, antisocial behavior, or externally imposed removal from school.

#### 3.6.3 Functional assessment tools

A central empirical referent of SRB in the literature is functional assessment. Kearney and Silverman (39) introduced the School Refusal Assessment Scale (SRAS) to identify the maintaining variables of school refusal behavior, and Kearney (14) later revised this approach in the School Refusal Assessment Scale–Revised (SRAS-R). These tools are designed to assess whether school refusal is primarily maintained by avoidance of school-based negative affect, escape from social or evaluative situations, pursuit of attention from significant others, or access to tangible reinforcement outside school (14, 39). Because functional heterogeneity is one of the defining attributes of SRB, these scales are particularly important empirical referents in both research and clinical assessment.

#### 3.6.4 Multi-informant and contextual referents

Finally, SRB is often identified not only through student self-report, but also through information provided by parents, teachers, and school staff (9). Such multi-informant data help clarify whether attendance difficulty is accompanied by parental awareness, school-related distress, peer difficulties, family conflict, or school-contextual stressors. This type of contextual information is especially important for distinguishing SRB from other forms of school attendance problems, such as truancy, school withdrawal, and school exclusion.

Taken together, these findings suggest that the empirical referents of SRB are best understood as a combination of behavioral, emotional, functional, and contextual indicators. In other words, SRB is not adequately identified by school absence alone. Rather, it becomes empirically recognizable when patterns of attendance difficulty are interpreted alongside school-related distress, functional motivation, and information from relevant adults and school systems. The conceptual relationships identified in the present analysis are summarized in **Figure 2**.

**Figure 2:**
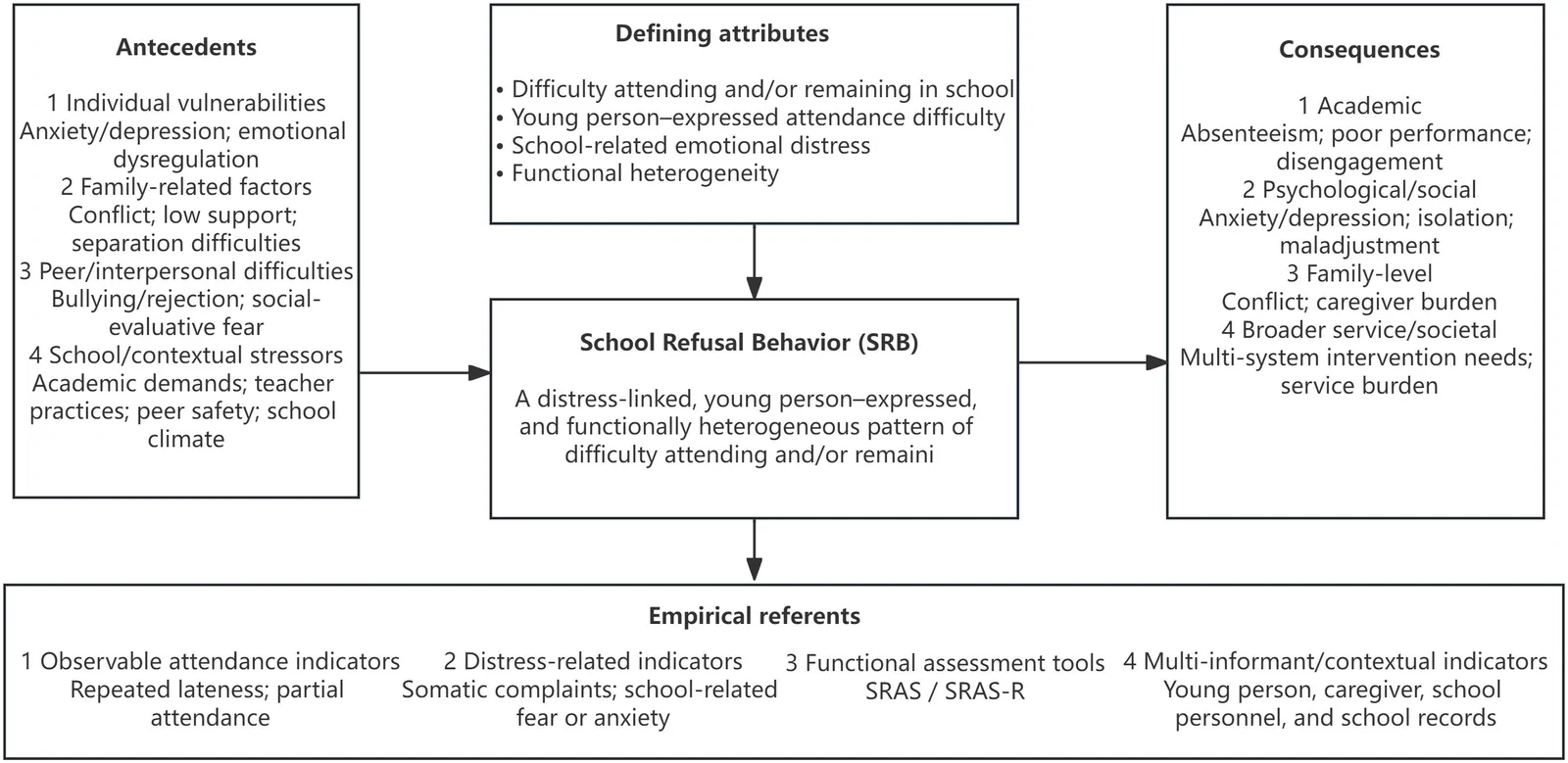
Conceptual framework of school refusal behavior (SRB).

### 3.7 Integrative and operational definition

Based on the defining attributes and empirical referents identified in this analysis, school refusal behavior (SRB) is operationally defined as a persistent or recurrent, youth-centered pattern of difficulty initiating, sustaining, or completing school attendance or participation. It may be manifested as repeated lateness, difficulty entering the school or classroom, partial attendance, requests to leave school early, intermittent absence, or prolonged nonattendance. These attendance difficulties are associated with aversive school-related experiences or emotional distress and may be maintained by one or more avoidance- or reinforcement-related functions.

In research and practice, identification of SRB should not rely on absence frequency alone. It requires consideration of four complementary sources of evidence: observable attendance patterns; emotional, behavioral, or somatic responses associated with school attendance; the functions maintaining the attendance difficulty; and contextual information obtained from the young person, caregivers, and, where appropriate, school personnel. SRB should be differentiated from absence primarily attributable to an acute or chronic medical condition, parent-initiated school withdrawal, school-imposed exclusion, or truancy characterized predominantly by concealment and rule violation. However, medical, psychological, family, and school-related factors may coexist and should therefore be assessed rather than treated as mutually exclusive explanations.

## 4 Discussion

Using Walker and Avant’s concept analysis method, this study systematically examined the uses, defining attributes, antecedents, consequences, and empirical referents of school refusal behavior (SRB). Rather than prioritizing one explanatory perspective, this analysis integrates previous accounts of SRB by considering emotional distress, avoidance functions, observable attendance difficulties, and contextual influences within a single conceptual framework. The findings suggest that SRB is not simply “not going to school,” nor should it be equated with anxiety disorders, truancy, or general absenteeism. Rather, it is best understood as a youth-centered pattern of difficulty initiating, sustaining, or completing school attendance and participation, shaped by aversive school-related experiences and heterogeneous maintaining processes (23, 40).

First, this study emphasizes that the core of SRB is not simply whether a student is absent, but whether the child or adolescent is already struggling to enter, sustain, or tolerate participation in school. School participation should therefore be viewed as a continuum rather than as a binary state of presence or absence. Attendance data may identify whether a student was physically present, but they do not capture whether the student could enter the classroom, remain for the full day, tolerate school demands, or participate meaningfully. Emerging forms of SRB may consequently become visible before conventional thresholds for excessive absence are reached. Recent clinical and parent-report research similarly suggests that lateness, early departure, intermittent absence, and classroom exit may precede prolonged nonattendance (41, 42), while young people may follow different trajectories of deterioration and recovery (43). Conceptually, SRB shifts attention from the final outcome of absence to the gradual erosion of the young person’s capacity to engage with school. This interpretation is consistent with broader SAPs frameworks that conceptualize attendance difficulties as multidimensional processes rather than isolated absence outcomes (9, 17).

Second, this study further suggests that the “refusal” in SRB should not be understood simply as active opposition or behavioral misconduct. In disciplinary language, refusal can imply a conscious decision to oppose expectations. However, attendance difficulty may be expressed through resistance or avoidance when the demands of school exceed the young person’s perceived or available capacity to manage associated emotional, somatic, interpersonal, or cognitive challenges. A child or adolescent may therefore struggle to attend or remain in school in circumstances where the available responses are constrained by distress and by the conditions they encounter within and around school. Importantly, understanding SRB in this way does not imply that non-attendance is simply irrational or maladaptive. Rather, avoidance may represent an attempt to manage experiences perceived as overwhelming, threatening, or difficult to tolerate within a particular personal and contextual situation. This interpretation is consistent with recent efforts to reframe apparent “school refusal” as “school can’t,” emphasizing the interaction between young people’s capacity to manage school-related demands and the conditions in which those demands are experienced, rather than willful noncompliance (44). Such a perspective reduces the risk of moralizing the behavior before its meaning and function have been understood. At the same time, SRB should not be equated with a specific mental disorder. Anxiety, depression, somatic distress, and emotional dysregulation are frequently associated with SRB, but the behavior itself is not a diagnostic category. Fear, anxiety, and phobia should also be distinguished conceptually. Fear refers to a response to a perceived immediate threat, whereas anxiety involves anticipation of possible future threat and may range from normative apprehension to clinically significant symptoms. Phobia, by contrast, refers to persistent and disproportionate fear or anxiety associated with clinically significant distress or impairment (45). School-related fear or anxiety should therefore not automatically be regarded as psychopathological, as it may reflect responses to academic, interpersonal, or environmental threats. Similarly, the historical term “school phobia” should not be treated as synonymous with SRB or as implying that school-related distress necessarily constitutes a phobic disorder (22, 23). Earlier clinical work likewise showed that categorical diagnoses alone are insufficient to explain attendance difficulty and should be considered together with functional analysis and individualized formulation (46). SRB occupies an intermediate position between observable attendance behavior and clinical interpretation: it identifies meaningful impairment while leaving open which psychological, developmental, medical, family, peer, or school-related processes are most relevant (16, 46). This position may prevent both under-recognition, in which SRB is dismissed as ordinary absenteeism, and over-pathologization, in which it is automatically treated as evidence of anxiety or depression (23, 35).

A central conceptual issue concerns the relationship between emotional distress and functional heterogeneity. Traditional descriptions of school refusal have emphasized emotional distress, parental awareness, and limited severe antisocial behavior (11), whereas the functional model includes both negatively and positively reinforced patterns (47). These perspectives overlap but are not fully interchangeable. The present analysis clarifies this tension by treating emotional distress and maintaining function as responses to different conceptual questions. Emotional distress concerns how school attendance is experienced or expressed by the young person, whereas function concerns what is avoided, obtained, or regulated through the behavior and therefore helps explain its persistence. Emotional distress appears especially prominent when attendance difficulty is maintained by avoidance of negative affect or escape from social and evaluative situations (14), but its form and visibility may vary across profiles. Some young people may report overt anxiety, whereas others may mainly show irritability, somatic complaints, attachment-seeking behavior, or attraction to activities outside school (48). Accordingly, variation in overt distress does not eliminate the need to examine the young person’s aversive experience, just as the presence of distress does not by itself explain the function maintaining the behavior. The findings therefore support a layered understanding in which emotionally based school refusal represents an important presentation within the broader SRB construct, while functional assessment explains heterogeneity in its maintenance (10). Future studies should specify which level is being examined rather than treating related terms as interchangeable.

Functional heterogeneity offers another important lens for understanding SRB. Observable attendance patterns cannot, by themselves, explain why the behavior persists. Two students may show similar absence levels while differing substantially in the consequences maintaining their behavior (27). This supports a distinction between behavioral topography and function: topography concerns what the attendance difficulty looks like, whereas function concerns what the young person avoids, obtains, or regulates through the behavior (14). Recent profile-based studies indicate that heterogeneity remains evident even among students whose difficulties are broadly maintained by negative reinforcement, with subgroups differing in perceived health, resilience, and psychosocial functioning (49). Interventions based only on visible absence may therefore fail to address the relevant maintaining process. Functional explanations help clarify why attendance difficulties persist, but they do not fully explain how such difficulties emerge within particular developmental and educational contexts. The individual, family, peer, and school domains identified in this analysis should not be interpreted merely as separate lists of risk factors, nor should attendance difficulty be attributed solely to characteristics of the young person. Rather, these domains represent interacting systems through which school attendance becomes more or less difficult to sustain. Individual emotional and regulatory characteristics may influence how young people perceive and respond to academic or interpersonal stressors (28, 30); family responses may buffer distress or unintentionally reinforce avoidance (15, 31); and school practices may influence whether attendance demands are experienced as manageable, supportive, or overwhelming (33, 34). Responsibility for understanding and responding to attendance difficulty should therefore not rest solely with the young person or family; schools and school leaders also have a role in identifying and addressing modifiable psychosocial and organizational barriers to participation. The same factor may also change its role over time. Family accommodation may initially be protective but may later become associated with continued nonattendance (50), while academic difficulty may precede SRB and may become more pronounced as absence accumulates (4). Distinct multi-year trajectories support this temporal view, showing that some students recover rapidly whereas others deteriorate and require prolonged recovery (43). By considering functional processes together with emotional and contextual influences, this analysis places avoidance mechanisms within the broader conditions under which school participation becomes difficult to sustain. Antecedents, maintaining mechanisms, and consequences are therefore dynamically interconnected, reflecting the interaction between young people’s experiences and the relational and educational environments in which school participation occurs.

The findings of this study indicate that the consequences associated with SRB may become interconnected over time, although this process is unlikely to follow a uniform trajectory. In the short term, avoidance may provide relief from aversive school-related experiences, particularly when attendance is perceived as overwhelming or difficult to sustain. When underlying difficulties remain unresolved, however, persistent non-attendance may coexist with reduced opportunities for academic participation, peer reconnection, and emotional adjustment. Some longitudinal evidence supports the possibility of reciprocal processes. For example, school refusal and depressive symptoms have shown prospective bidirectional associations, indicating that attendance difficulty and emotional distress may become mutually reinforcing for some adolescents (51). This possibility should not, however, be interpreted as evidence that non-attendance itself inevitably worsens SRB. Trajectory research shows substantial heterogeneity, including rapid recovery, intermittent absence, prolonged recovery, and gradual or rapid decline (43), while longer-term follow-up has likewise shown considerable variation in functional outcomes (52). Thus, a worsening cycle may characterize some trajectories, but it should not be assumed to represent the natural course of SRB for all young people. The meaning and consequences of non-attendance are likely to depend on its function, duration, and the individual, family, peer, and school conditions surrounding it. School re-entry should therefore not be conceptualized simply as reversing absence, but as a process that also requires attention to the conditions contributing to attendance difficulty.

Taken together, the proposed definition brings together the observable pattern of attendance difficulty, the young person’s school-related experiences, the functions maintaining the behavior, and the wider family, peer, and school context. These elements are closely related, but they answer different questions: attendance difficulty shows what is happening, emotional and somatic responses show how the difficulty is experienced, functional assessment helps explain why it persists, and contextual information helps determine how it develops and how it differs from other attendance problems. This framework therefore provides a clearer basis for identifying and understanding SRB without reducing it to a single symptom, diagnosis, cause, or attendance threshold. Because the definition was derived from conceptual analysis, it should be regarded as a framework for further testing rather than a diagnostic rule. Future research should examine its applicability across developmental stages, cultural settings, clinical populations, and educational systems.

### 4.1 Implications

The findings of this concept analysis have practical implications for the early identification and management of school refusal behavior (SRB) across child and adolescent mental health, pediatric, primary care, and school-based settings. Children and adolescents with emerging attendance difficulties may initially present with recurrent headaches, abdominal pain, fatigue, sleep disturbance, anxiety, low mood, irritability, or other symptoms that become more pronounced before school. These presentations may be interpreted as isolated physical or emotional complaints unless school attendance and participation are specifically explored. Professionals should therefore consider changes in attendance alongside emotional and somatic symptoms, including repeated lateness, partial attendance, difficulty entering or remaining in class, early departure, and increasing distress on school mornings. Recognizing these patterns may allow support to begin before attendance difficulties progress to prolonged nonattendance.

Assessment should draw on multiple sources rather than relying on absence records or a single informant. The young person’s account is essential for understanding school-related experiences, distress, perceived safety, and reasons for avoidance, while caregivers may provide information about morning difficulties, physical complaints, family responses, and changes over time. With appropriate consent, information from school personnel may clarify classroom participation, peer relationships, academic pressures, and school-based triggers. Differences among informants may reflect variation across settings rather than inaccurate reporting. Assessment should also examine co-occurring anxiety, depressive symptoms, somatic distress, emotional dysregulation, developmental difficulties, and the family and school conditions that may shape the young person’s experience of attendance demands. SRB should not itself be treated as a psychiatric diagnosis; rather, these findings should inform an individualized formulation of how attendance difficulties develop and what maintains them. A multi-informant approach can also help distinguish SRB from absence primarily related to physical illness, truancy, parent-initiated withdrawal, or school-imposed exclusion (9).

When SRB is persistent, causes marked functional impairment, or occurs alongside clinically significant mental health symptoms, timely referral to child and adolescent mental health services may be required. Coordination among clinicians, families, and schools is important because fragmented responses may lead to repeated assessment without a shared understanding or intervention plan. Collaborative care should identify the young person’s main difficulties, clarify appropriate school support, consider contextual barriers to participation, and establish realistic steps toward gradual re-engagement (53–55). School leaders and relevant staff should be active participants in this process, with responsibility for reviewing modifiable school conditions, including academic demands, teacher practices, peer safety, and the availability of appropriate supports or accommodations. Families should also be active participants in this process and supported to reduce blame, validate distress, and respond consistently without unintentionally reinforcing prolonged avoidance. Progress should be evaluated not only through attendance, but also through emotional functioning, sustained classroom participation, and the young person’s capacity to participate in school within the available academic and interpersonal supports (50).

### 4.2 Limitations

This study has several limitations. First, the analysis was restricted to English-language literature, which may have excluded relevant conceptualizations from other linguistic or cultural contexts. Second, because terms such as school refusal, school refusal behavior, emotionally based school avoidance, and broader school attendance problems are not used consistently across disciplines and countries, some degree of interpretive judgment was unavoidable in distinguishing conceptually adjacent phenomena. Finally, as a concept analysis, the present study was intended to clarify the meaning and boundaries of SRB rather than to test causal relationships or evaluate intervention effectiveness directly.

## 5 Conclusion

In sum, this concept analysis clarifies school refusal behavior (SRB) as a persistent or recurrent, youth-centered pattern of difficulty initiating, sustaining, or completing school attendance and participation. These difficulties are associated with aversive school-related experiences or emotional distress and may be maintained by heterogeneous avoidance- or reinforcement-related functions within family, peer, and school contexts. By distinguishing SRB from truancy, school withdrawal, and school exclusion without equating it with a psychiatric diagnosis, the proposed definition provides a clearer conceptual basis for early recognition, differential assessment, individualized formulation, and coordinated intervention.
